# Views and Experiences of Adults who are Overweight and Obese on the Barriers and Facilitators to Weight Loss in Southeast Brazil: A Qualitative Study

**DOI:** 10.1080/17482631.2020.1852705

**Published:** 2020-11-29

**Authors:** Caroline Morgan, Gilles de Wildt, Renata Billion Ruiz Prado, Nisha Thanikachalam, Marcos Virmond, Ruth Riley

**Affiliations:** a College of Medical and Dental Sciences, University of Birmingham, Birmingham, UK; b Research Institute, Instituto Lauro De Souza Lima, Bauru, Brazil

**Keywords:** Barriers, Brazil, facilitators, obesity, qualitative, weight loss

## Abstract

**Background**: Obesity in Brazil is increasing with 54% of the Brazilian population being overweight, of which 20% is obese. Obesity is a risk factor for non-communicable diseases such as cardiovascular disease: the leading cause of mortality in Brazil. This study aims to identify the barriers and facilitators to weight loss as perceived by patients with a view to reducing the burden of obesity-related diseases on patients and healthcare services.

**Methods**: Fifteen qualitative, semi-structured, in-depth interviews were conducted in the preventive medicine department in a private health clinic in Bauru, Southeast Brazil. Inductive thematic analysis was conducted.

**Results**: The barriers and facilitators were classified into three themes: lifestyle, motivation and education.

Barriers include cost of a healthy lifestyle, time management, personal safety, mobility, junk food advertising, sustaining weight loss, mental health, lack of support and health education.

Facilitators include change in eating habits, sleep quality, cooperative food networks, access to the multidisciplinary team and expert patients as health educators.

**Conclusion**: Expert patients should be utilized as an education method, as they increase motivation, promote the facilitators and provide realistic expectations of the weight loss process. Barriers such as junk food advertising and accessibility to treatment need to be addressed.

**Abbreviations**:

BMI: Body Mass Index; NCD: Non-Communicable Disease; SUS: Sistema Único de Saúde; WHO: World Health Organization

## Introduction

Obesity is a growing problem globally, with 1.9 billion adults in the world being overweight, of which 650 million are obese (World Health Organization, 2018a). The World Health Organization (WHO) defines overweight as a Body Mass Index (BMI) greater than 25 kg/m^2^, and obesity is greater than 30 kg/m^2^ (World Health Organisation, 1995). In Brazil, 54.2% of the population is overweight, of which 20.1% is obese (World Health Organization, 2016). The obesity rate increased by 50% between 2006 and 2012 and it is predicted that by 2022, almost two-thirds of the population will be overweight and a quarter obese (Malta et al., 2014). Brazil performs the second highest number of bariatric surgeries (0.04334% of the total population) following the USA (0.044% of the total population) (Angrisani et al., 2015; Ramos, 2014). In comparison, the rate of bariatric surgeries in the UK population is 0.0087% (Angrisani et al., 2015). The rise in prevalence of obesity is attributed to cheap high caloric foods, increased prices on nutritious foods, physical inactivity and stress (Monteiro et al., 2007).

Obesity is a major risk factor for non-communicable diseases (NCDs) such as cardiovascular disease, non-insulin dependent diabetes mellitus, musculoskeletal disease and cancer (Guh et al., 2009). NCDs cause 74% of deaths in Brazil, with cardiovascular disease being the top cause of mortality for the past 50 years (Ribeiro et al., 2016; World Health Organization, 2018b). Additionally, obesity can severely impact patients psychologically, including body image dissatisfaction, low mood and self-esteem. The media presents negative messages about obesity which results in anti-fat bias causing stigma and discrimination, for example, in education, employment, health and social settings (Schwartz & Brownell, 2004). An increased concern for body image in Brazil may be attributed to the hot climate and many beaches encouraging the population to wear body-exposing light clothing, therefore causing increased body image dissatisfaction (Silva et al., 2011).

Weight loss can improve cardiovascular risk factors such as hypertension, type 2 diabetes mellitus and metabolic syndrome, with clinically significant weight loss (>5% of baseline body weight) being most effective in reducing cardiovascular disease and type 2 diabetes mellitus risk factors (Wing et al., 2011). Weight loss can also reduce the economic burden of obesity and obesity-related diseases which currently costs 2.4% of GDP in Brazil (Dobbs et al., 2014). An estimation of US$330 billion will be spent on obesity between 2010 and 2050; however, US$27 billion can be saved within the 40 years if the obesity rate decreases by 1% (Rtveladze et al., 2013).

Weight loss strategies include caloric restriction and physical activity. Public health recommendations on physical activity have shown to be only effective with caloric restriction, however high volume aerobic exercise achieves clinically significant weight loss without caloric restriction (Swift et al., 2014). Pharmacotherapy is another effective weight loss method, however if lifestyle changes or pharmacotherapy strategies fail, weight loss surgery is an effective weight loss method and is associated with improved quality of life and remission of comorbidities (Colquitt et al., 2014; Neovius et al., 2008). However weight loss surgery is associated with more adverse events and complications such as infection and hypoglycaemia compared to non-surgical therapy (Colquitt et al., 2014). Additionally, the literature have reported that a multidisciplinary approach to weight loss is more effective than an individual physician approach (Feigenbaum et al., 2005).

Despite the many weight loss strategies implemented in Brazil, obesity rates remain high (Jaime et al., 2013). Qualitative studies have explored barriers and facilitators to weight loss in North America and Europe (Alm et al., 2008; Gupta, 2014; Hammarström et al., 2014; McVay et al., 2018; Metzgar et al., 2015). However, qualitative studies have reported cultural variations in views and experiences to weight loss, suggesting the possibility of variation in the Brazilian context (Bramble et al., 2009). One qualitative study in Northeast Brazil reported difficulty in diet control, lack of motivation, fatigue, medical conditions preventing physical activity, financial difficulty, occupation and influence of healthcare professionals and family members as factors affecting weight loss (S Palmeira et al., 2016). However, no qualitative studies on the barriers and facilitators to weight loss have been conducted in Southeast Brazil which is more socioeconomically and industrially developed than the Northeast (Porsse et al., 2012). One study in the USA showed that disparities in socioeconomic status influences access to weight loss treatment (Tsai et al., 2009). Socioeconomic status has an impact on obesity by the increase in availability of obesogenic food, thus increasing the risk of obesity. However, it also provides greater access to information, for example, through the media which may be a facilitator to weight loss (Monteiro et al., 2000). Therefore, this study aims to identify barriers and facilitators to weight loss in the more socioeconomically developed and more obese Southeast Brazil and address any changes required to reduce the obesity rates and burden of NCDs on patient health and healthcare services.

## Methods

### Study design and sampling

A qualitative approach was used to focus on context and variation of views and experiences which are unknown at the beginning of the investigation and difficult to quantify. The setting was in the preventive medicine department of a private health clinic providing weight counselling in the city of Bauru, Sao Paulo State, Southeast Brazil. The data were collected over 4 weeks in February 2019. The setting at a specialist weight counselling clinic allowed for purposive sampling to be conducted, recruiting information-rich participants with experience of the barriers and facilitators to weight loss.

### Participants and recruitment

Participants were required to meet the inclusion criteria to participate in the study. Inclusion criteria were patients: registered at the health centre, over the age of 18, males and females, BMI greater than 25 kg/m^2^, able to give informed consent, able to speak Portuguese or English fluently. Exclusion criteria was impaired cognition which was assessed during the consultation during recruitment. If cognitive impairment was suspected, the recruiter referred to the “six-item cognitive impairment test” to assess cognitive status (Gale & Larner, 2017).

Eligible participants were identified by the nutritionist, psychologist, doctor or nurse during weight counselling consultations at the health clinic. Interested participants met CM (primary researcher) who explained the study using the participant information sheet. The reason for patients’ refusal to participate in the study was their lack of time to conduct the interviews.

### Ethical considerations

The research was approved by the BMedSci Population Sciences and Humanities Internal Ethics Review Committee at the University of Birmingham, Scientific Committee and Ethics Committee at Instituto Lauro de Souza Lima. All participants signed an informed consent form before the interviews. Data were stored according to the University of Birmingham data protection policy (University of Birmingham, 2018).

### Data collection

Data were collected using in-depth semi-structured interviews. Individual interviews were used to allow rapport and trust to be built, especially due to the sensitivity of certain topics such as body image which may be addressed during the interviews. The interviews were conducted in Brazilian Portuguese and English using real-time interpretation between the primary researcher, interpreter and participant. CM is female and conducted the interviews and took field notes, with the presence of an interpreter to translate the interviews from Brazilian Portuguese into English. The interpreters were MV (male) and RP (female) who are researchers involved in the study and the third interpreter was another researcher (female) at Instituto Lauro de Souza Lima. CM briefed the interpreters about the aims of the study, the importance of open questions, any issues from previous interviews and the interpreters practiced asking the interview questions to minimize any inconsistencies in data collection. The use of an interpreter allowed for the primary researcher to have more control over data collection and flexibility in follow-up questions. An iterative approach was used to allow the inclusion of novel ideas until data saturation was achieved.

The interviews were conducted following a semi-structured topic guide (Table 1) developed from the aims of the study and review of previous qualitative studies (S Palmeira et al., 2016; CS Palmeira et al., 2013). The use of open questions allowed for flexibility in answers and closed questions were used for further clarification and in-depth exploration. A pilot phase was conducted on the first day of interviews and following feedback from the participants and discussion between the researchers, minor adjustments to the demographic form and probing questions in the topic guide were made.

**Table 1.** Interview topic guide

Topic	Question
Beliefs	What is your understanding of obesity?
	What is your view on being obese?
	Are there any advantages or disadvantages to obesity?
Weight loss	What is your experience with weight loss?
	What do you know about weight loss?
	What are the barriers to weight loss?
	What are the facilitators to weight loss?
Future steps	What can be done in the long term to help with weight loss?

The interviews were audio-recorded, and the spoken Brazilian Portuguese was transcribed and translated into English by independent interpreters who were three local university students training to be professional interpreters. The English transcripts were revised by a second interpreter to ensure accurate English interpretation. The cultural context and colloquialisms were described in note form by the interpreters. Minimal discrepancies in interpretation were noted and therefore minimal changes were made following discussion with the interpreters.

The average length of the interviews was 27 minutes. The interviews were conducted in the same private consultation room at the health clinic to ensure consistency in the environment for all interviews.

### Data analysis

**Figure 1.**
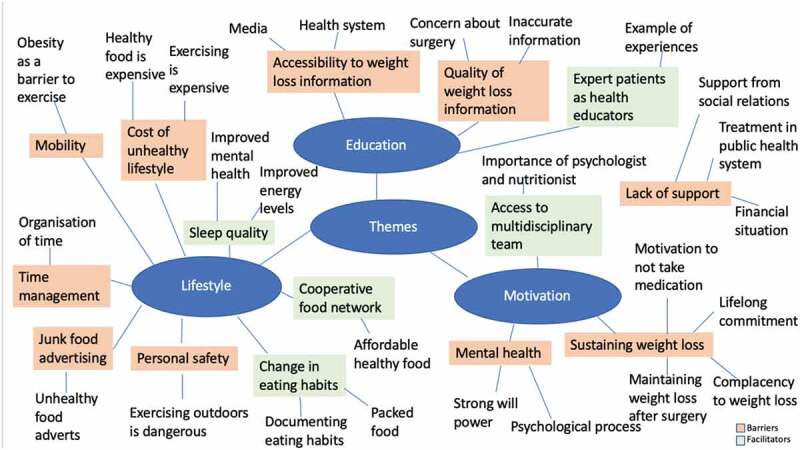
Thematic mindmap of themes and sub-themes

Data were analysed using inductive, semantic thematic analysis, meaning themes were developed from the explicit meanings from the data. The analysis was conducted following Braun and Clarke’s six steps for thematic analysis (Braun & Clarke, 2006). This allowed for flexibility in the analysis and theme development. Firstly, all of the transcripts were read several times to immerse CM with the data. Next, initial codes were generated from phrases and sentences using support from the NVivo 12 software. The codes were named to reflect the views and experiences described by the participants. The codes were revised by CM by re-reading the transcripts to ensure they accurately described the data and to ensure no codes had been missed. Thirdly, similar codes were grouped together by creating thematic mind-maps (Figure 1) to represent the sub-themes and subsequently subthemes were grouped together into the key themes. The themes and subthemes were reviewed and refined in two cycles: first cycle by reading quotes from the data and the second cycle by reading the entire data set to ensure the themes accurately described the data.

To ensure validity of the data, a second qualitative researcher (NT) independently coded five of the most data-rich transcripts for analysis triangulation and to reduce research bias. Similarities and differences were noted, and any differences were discussed between the researchers until a consensus was reached. A reflexive approach to analysis was used, where notes were made throughout the analytical process to assess the potential impact of researcher bias. The study was reported using the “Consolidated criteria for reporting qualitative research (COREQ)” checklist (Tong et al., 2007).

## Results

### Participants

A total of 15 patients participated in the study and 13 patients declined to participate. 12 of the participants were female and 3 were male. The average age was 43 years and the average BMI was 37.4 kg/m^2^. The demographic information is summarized in Table 2.

**Table 2.** Demographic information of the participants

Participant ID	Sex	Age range (years)	Body Mass Index (kg/m^2^)	Ethnicity	Other health conditions	Highest level of education	Monthly household income (US$)	Employed	Marital status
P1	Female	30–39	44.5	White	No	University	790–1,300	Yes	Cohabiting
P2	Female	30–39	38.9	Black	Yes	High School	530–789	Yes	Married
P3	Female	40–49	30.2	Brown	Yes	High School	<265	No	Married
P4	Male	40–49	35.8	White	Yes	High school	530–789	Yes	Married
P5	Female	50–59	37.3	White	Yes	College	530–789	No	Married
P6	Male	30–39	39.2	Black	Yes	University	790–1,300	Yes	Married
P7	Female	30–39	37.6	White	No	High School	530–789	Yes	Cohabiting
P8	Female	40–49	25.2	White	Yes	Post-graduate	530–789	Yes	Married
P9	Male	30–39	33.9	White	Yes	Post-graduate	790–1,300	Yes	Married
P10	Female	40–49	44.1	White	Yes	High School	265–529	Yes	Married
P11	Female	40–49	41.2	Black	No	High School	265–529	Yes	Married
P12	Female	60–69	44.9	White	Yes	University	530–789	Yes	Divorced
P13	Female	30–39	27.6	Black	No	College	530–789	No	Married
P14	Female	40–49	47.3	White	Yes	University	530–789	Yes	Divorced
P15	Female	50–59	32.6	White	Yes	Post graduate	530–789	Yes	Single

### Findings

The paper reports the views and experiences of adults who are overweight and obese on the barriers and facilitators to weight loss in Southeast Brazil. The barriers and facilitators were classified into three key themes (World Health Organization, 2018a) lifestyle, (World Health Organisation, 1995) motivation and (World Health Organization, 2016) education. The barriers in lifestyle were divided into five subthemes: a) cost of a healthy lifestyle, b) time management, c) personal safety, d) mobility and e) junk food advertising. The barriers in the theme motivation were divided into three subthemes: f) sustaining weight loss, g) mental health, h) lack of support. The barriers in the theme education were divided into two subthemes: i) accessibility to weight loss information, j) quality of weight loss information. The facilitators in lifestyle were divided into three subthemes: a) change in eating habits, b) sleep quality, c) cooperative food network. The facilitators to motivation included one subtheme: d) access to the multidisciplinary team. Facilitators to education included one subtheme: e) expert patients as health educators.

### Barriers

#### Theme 1: lifestyle

##### Cost of a healthy lifestyle

All participants expressed that an unhealthy lifestyle which includes physical inactivity and unhealthy eating habits is easily accessible. High financial costs of a healthy lifestyle were expressed as a barrier by most participants, resulting in unhealthy lifestyle habits.

“*it’s expensive to be on a diet in Brazil. It is very expensive …. It’s cheaper to eat junk food … . becoming fat is cheap in Brazil; losing weight is expensive.” (P8)*

“I think that many people consider going to the gym, but think that it’s not affordable”(P1)

However, two participants believed that healthy food is more affordable than unhealthy food and it was rather the time and effort that was a barrier to preparing healthy meals.

“Today, healthy food is cheaper than unhealthy food.” (P2)

“I thought I didn’t have time to prepare it because if you buy fruits and vegetables, you have to buy them regularly; it is not something that lasts the whole month and you need time to prepare them” (P7)

##### Time management

Participants described the lack of time and rush of daily routine and occupation preventing the preparation of healthy meals and exercise, attributing to an unhealthy lifestyle.

“maybe this rush, lack of quality of life, you have to run from one job to another and it’s difficult to find the time to exercise, maybe this contributes as a barrier.” (P9)

Three participants discussed time-management methods to reduce the barrier to improving eating habits. One method was using alarms on their smartphone as a reminder to eat at regular intervals.

“I started to set an alarm on my cell phone because where I work, we are always in a hurry, so I don’t stop … . I work and then I end up forgetting to eat.” (P2)

##### Personal safety

Exercising outdoors could be a solution to the expensive cost of gyms; however, the lack of safety in exercising outdoors such as encountering dangerous people and avoiding the sun and heat was described as a barrier.

“When people say “if they can’t afford to go to the gym, they should do it outdoors”. I tried that, it’s dangerous. If you are going to exercise, you have to do it before 7 am because of the sun, or at the end of the afternoon, when the sun goes down. Both are dangerous because you might encounter dangerous people on the way.” (P13)

##### Mobility

Most participants expressed that obesity and obesity-related illnesses can cause mobility problems such as fatigue and joint problems which are a barrier to exercise and therefore contribute to an unhealthy lifestyle.

*“being heavy like I* *am, I* *don’t have the physique to move really fast, to do physical activities more frequently* *… Why? Because I* *can’t walk a* *lot, because my knees hurt” (P10)*

However, despite some participants developing obesity-related mobility problems and health issues, they described this as a motivator to lose weight to improve their quality of life.

*“I had inflammation on my foot due to my weight which was terrible, a* *horrible pain, and since we need our feet for everything, even for getting up, I* *said: ‘This can’t continue’. That’s what made me say: ‘Now it is all or nothing.’” (P11)*

##### Junk food advertising

Most participants described the easy accessibility of unhealthy food, including ready-meals, junk and processed food that are offered in the supermarkets and advertisements. One participant suggested that adverts for unhealthy food should be prohibited.

“‘*Ready-meals, ‘it tastes like homemade food. Your mom’s food.’ You see it, it is there on the adverts* *… … . Just as the cigarette adverts were prohibited* *… . we shouldn’t have alcoholic beverages, sodas, chips, and junk food adverts.” (P8)*

#### Theme 2: motivation

##### Sustaining weight loss

Participants emphasized that acceptance of a lifelong commitment to a change in habits is crucial to maintain weight loss. Some participants explained that a lack of commitment to habit and lifestyle change is a barrier to weight loss.

“you want to lose (weight), but you don’t want to let go of many habits and that messes with you so badly” (P11)

Some participants explained that successful weight loss could result in complacency and belief that they will not regain the weight. This consequently causes a loss in commitment to a healthier lifestyle.

*“It’s a* *joy at first, but then you say: “I lost weight, it’s good”, then you start gaining weight again, because you relax thinking “I did it, so I* *can do it again”, and you end up relaxing.” (P14)*

Participants explained that bariatric surgery is not the final solution to treat obesity, but rather the first step to weight loss and requires a commitment to attend follow-up appointments and follow a healthy lifestyle after surgery.

“All of them had the same point of view that the surgery was the first step because it is not the last step, it is the first one to lose weight.” (P8)

Additionally, many participants described the use of anti-obesity medication as ineffective in the long term since weight regain is common once the medication is stopped, discouraging their progress.

*“They had results. But all the diets that I* *have done were based on medication. So, when I* *stopped taking the meds, the obesity came back, sometimes even double of what it was before.” (P10)*

Some participants described their prior experiences of negative side effects from anti-obesity medication as a barrier to weight loss.

*“you become a* *hostage to them and they are very harmful, like, I* *took drugs to lose weight for many years and after some time I* *started having memory loss, even today, sometimes I* *don’t remember something, I* *can’t memorise words, insomnia, and also stress, it’s a* *drug that affects the nervous system, so you have to take other drugs to feel calmer. It’s like a* *bomb in your body, so after some time you don’t want that anymore, because you realise it’s very harmful.” (P11)*

On the other hand, some participants were unwilling to be medicine dependent due to their comorbidities, which increased motivation to lose weight.

*“I have never wanted to become medicine dependent. So, I* *started being worried about that.” (P6)*

##### Mental health

The relationship between mental health and weight loss was described as a barrier by most participants. They explained that conditions such as anxiety or depression could cause excessive comfort eating, preventing weight loss.

*“I think it is the compulsivity and anxiety. Every time I* *was nervous, I* *wanted to eat a* *sweet, so if I* *had something to do that demanded responsibility, a* *deadline, I* *couldn’t keep up with my diet* *… … Also, family issues. All the sadness, all the anguish also affected me. It was something that prevented me from eating properly” (P12)*

Some participants explained that obesity lowered self-esteem which consequently becomes a barrier to weight loss as patients are less motivated to lose weight or are too self-conscious to exercise in the gym.

*“When you are overweight, your self-esteem is low and you are like: ‘You are overweight.’ You think that you are going to the gym and people are going to stare at you. This is something that has blocked me from doing something I* *like.” (P1)*

##### Lack of support

All participants described the importance of support from family and social relations to lose weight. One participant described: *“family support, my spouse, for me, was important … my friends, at work, everybody encouraged me.”* (P8) Despite this support, participants described the social environment as a barrier to weight loss. For example, not all household members will decide to follow a diet, so two different meals are prepared in the house.

*“I think that the greatest difficulty is when people that live with you don’t have the same goal. It is difficult for a* *person to prepare something just for you* *… . I* *think this is the barrier in obesity.” (P1)*

One participant expressed their doubts in whether healthcare professionals understood the struggles that obese patients face with weight loss which can lead to their words of encouragement being interpreted as criticism rather than motivation.

*“Most professionals see it this way, as laziness: ’You don’t do it, because you like to stay lying down all* *day doing nothing.’* *… … So, your success so far was lost, because the person judges you as lazy, like you didn’t try hard enough, so I* *think this is the worst part for the person who is really trying, not seeing any results at that moment and is criticized.” (P14)*

Some participants have described the lack of certain healthcare professionals such as nutritionists in the public healthcare system, which participants emphasized were crucial to facilitating the weight loss process.

*“The Public Healthcare System doesn’t have a* *nutritionist in all of its health care centers. There isn’t someone to help the population.” (P13)*

The participants discussed the barriers to accessing bariatric surgery in the Sistema Único de Saúde (SUS)- public funded health system such as the lengthy referral time, the criteria to access it and that many people within the population are unaware that it is available in the SUS.

*“Today, a* *lot of people don’t know that they have it on SUS* *… . but it already involves a* *lot of bureaucracy just to get that referral and it takes a* *lot of time. I* *don’t think it is something that happens quickly.” (P4)*

Lack of financial support for example, to access weight loss treatments such as a weight loss programme or bariatric surgery can be a barrier as all participants accessed treatment through private health insurance, since they perceived it to be less accessible through the SUS.

*“The financial aspect* *… I* *think that it is critical for the ones who want to lose weight* *… . My healthcare insurance covered everything. What about those who don’t have it? This surgery costs R$ 50,000 (US$13,000) in Brazil, considering doctors, hospital, it is R$ 50,000 (US$13,000).” (P8)*

Some participants suggested that more support from the government for example, providing exercise facilities for those that cannot afford it can reduce the barriers to uptake of a healthy lifestyle.

*“Offer to the ones that can’t afford swimming classes, gyms. Not everything should depend on the people’s income. So, I* *think that the city should think about it.” (P12)*

#### Theme 3: education

##### Accessibility to weight loss information

All participants had accessed obesity and weight loss information through the media: “*Magazines, internet, emails about diets, weight loss, also on Facebook.”* (P2) and described them as facilitators. However, some participants believed that these sources through the media are less reliable and that the most reliable information is through the health system. One participant suggested the provision of social assistance by a healthcare professional for guidance on weight loss.

*“I think it would be ideal to be inside the health system* *… . Because, for example, for me to get where I* *am today, I* *went after it because of my health, that was when the doctor said I* *had to take care of myself, ‘you need to lose weight.’ So, inside a* *health center today, or social assistance.” (P4)*

Some participants explained that the lack of accessibility to reliable weight loss sources can reduce awareness of the health consequences of obesity in the population, resulting in a complacent attitude towards obesity.

“I think people should be more aware of obesity because sometimes people think they are fat and that it is harmless there won’t be any problems in the future.” (P1)

##### Quality of weight loss information

The participants discussed the importance of the quality of information provided at both the individual and societal level. Inaccurate information can result in avoidance of specific beneficial weight loss treatments such as bariatric surgery due to either the patient’s or the family’s concern.

*“I had never thought about bariatric surgery, I* *used to say: ‘No way that I’m going to undergo such a* *risky surgery’, because the information I* *had was that some people ended up with mental problems, people who didn’t follow the instructions and got sick, etc., so I* *didn’t have factual information about what bariatric surgery was.” (P11)*

Participants explained that the lack of quality education on weight loss methods can result in a long period of unsuccessful weight loss.

“It took me more than ten years to realise what is really necessary to lose weight.” (P11)

Therefore, it is crucial to target these myths about weight loss methods and surgery by providing good quality and accurate information in weight loss education.

*(Researcher): “What would work to promote weight loss?”*

“I think information, but factual information.” (P11)

### Facilitators

#### Theme 1: lifestyle

##### Change in eating habits

The participants discussed their experiences with facilitators to a healthy lifestyle for weight loss, which included changing eating habits such as preparing healthier meals, eating at regular intervals, eating smaller portions and chewing slowly. One participant explained the importance of gradually changing eating habits as extreme changes such as completely restricting certain foods could lead to cravings, and consequently weight regain.

*“don’t restrict everything* *… … because if you restrict something you like a* *lot, you will follow it for some time, but not for a* *long time, because you can’t continue without it and you will want to eat it double.” (P14)*

Packed meals prepared at home have been described as a facilitator to weight loss and a healthier lifestyle. It also prevents social exclusion caused by patients avoiding social gatherings due to the temptation of unhealthy food. However, it has been described as something that requires more effort and time and may not be considered important for young people.

*“I went out with my friends. We even went to a* *pizza place, but I* *took my packed snack.” (P8)*

*“when you are 15/16 years old, you just don’t think about packing your food and taking it to your workplace. Today, I* *see that I* *could have done it.” (P1)*

One participant described the benefit of documenting eating habits with a food diary as a facilitator to lose weight.

*“I started keeping a* *diary with what I* *eat every three hours, so I* *can see: “Wow* *… It has been four hours since the last time I* *ate.” Because you go there to register the time and you see the last time you ate* *… . I* *started feeling less hungry after I* *started doing this because now I’m not eating when I’m hungry.” (P1)*

##### Sleep quality

Sleep quality has been described as a lifestyle factor that affects weight loss and is linked to mental health conditions such as anxiety. Good sleep quality can improve energy levels needed for exercise, preparing healthy meals and healthy eating habits.

*“It (controlling anxiety) improves the quality of sleep. Now, I’m not hungry, you eat without being hungry* *… any kind of food. So, when I’m fine, I* *don’t seek something to eat, I* *have a* *better night sleep, I* *can rest, I* *wake up feeling more willing.” (P15)*

##### Cooperative food network

One participant suggested using abandoned plots as local gardens to cultivate fruit and vegetables which could be sold within the community at a low price to promote healthy eating habits.

*“having public gardens to cultivate food, that would be cheaper because it’s expensive* *… . the city could sponsor the producers, the neighbourhood.* *… . so we can have a* *garden and sell things for the residents for a* *symbolic price. So, every* *day you go there and there is lettuce, tomatoes, eggplant, peppers* *…. ”(P8)*

#### Theme 2: motivation

##### Access to the multidisciplinary team

Participants described the multidisciplinary approach to weight loss counselling as a facilitator. This includes doctors encouraging weight loss and psychologists and nutritionists providing guidance and follow-up appointments. This multidisciplinary approach is provided in weight loss programmes which most participants have participated in and described as a facilitator to weight loss.

*“The psychologist helps, the psychiatrist clarifies, the nutritionist guides you, helps and shows you the way, so putting all of this together, you have the strength, the will to lose weight. I* *think that is more complete.” (P11)*

Some participants described weight loss as a psychological process; it is crucial for the mind to change to see a physical change in weight loss.

*“My experience is a* *more psychological process than physical* *… .The figure changes, but the mind changes even more. If the mind doesn’t change, the body doesn’t either. ”(P8)*

This emphasized the importance of the psychologist in the multidisciplinary team as they can treat any psychological or emotional conditions, resulting in strong will power which was emphasized as an important facilitator to remain motivated to weight loss and to overcome the battle that patients face with the temptation to break their diet.

*“it doesn’t matter what is going on around you because you made a* *commitment. This is when the psychologist acts. They help us to know ourselves, realise how important we are.” (P7)*

#### Theme 3: education

##### Expert patients as health educators

The participants discussed the impact of social capital in health promotion and education. Three participants attended lectures in the community on obesity and weight loss. These lectures were conducted by healthcare professionals and other patients who have experienced weight loss (expert patients). The healthcare professionals provided guidance about weight loss methods and health consequences of obesity and expert patients shared their experiences to be used as an example to others.

*“I told him that I* *was interested in doing bariatric surgery because of the talks I* *had seen him do* *… … they (expert patients) said how they lost weight and how much weight they lost. And there are doctors there too, they answer our questions.” (P4)*

Expert patients were described as beneficial educators as participants can visualize the weight loss transformation, either from looking at picture evidence or by watching them during the process. They can also learn about the weight loss benefits the expert patients have experienced which inspires the patients to lose weight.

*“There is a* *lady who works in the same place I* *do. She used to be way fatter than I* *am, and she had the surgery with the same doctor that I* *am going to have. And, today, she is really thin, her health is good, she stopped taking all her blood pressure medication, and now she just needs to maintain the surgery, with meds and all* *… . that helped me. You know? It motivated me because she is* *… .* *… . a* *living example. I* *saw something that was worthwhile* *… I* *got inspired.” (P10)*

Most participants accessed weight loss information from expert patients through social and professional relations, so participants suggested that the experiences of expert patients are shared on the internet, so they are widely available and used as the main source of health information.

*“a video in which you talk about your experience, what you have done, your before and after. ‘My life was like this.’ Make a* *video to explain it. On social media, Facebook, Instagram. Today I* *think this is what people are looking for* *… . a* *person who has gone through that to tell their story.” (P1)*

## Discussion

### Barriers

Weight loss and maintenance rely on lifestyle factors such as healthy eating and exercise. Participants described this as less accessible due to the expensive cost of healthy food and gyms in Brazil, supporting the findings from Palmeira et al (S Palmeira et al., 2016). The Brazilian government made commitments to the “United Nations Decade of Action on Nutrition 2016–2025” which included a policy to reduce the tax on fresh foods and provide cash transfers to low-income families to buy fresh produce (World Health Organisation, 2017). These commitments were to be achieved by 2019; however, most of the participants in the study (which was conducted in February 2019) still believed that healthy food was more expensive than junk food. However, two participants viewed healthy food as affordable and believed it was rather the time and effort required to prepare healthy meals that were the barriers. Most participants agreed that time restrictions due to occupation and daily routine were a barrier to preparing healthy meals and exercising, supporting previous literature (Morais et al., 2010; S Palmeira et al., 2016). The mixed response to affordability of healthy food suggests the need for increased advertisements for affordable and less time-consuming healthy meals.

The Brazilian government launched a strategic plan in 2011 to reduce NCDs which involved promoting physical activity through building public spaces for physical and leisure activities, building healthy urban spaces such as sidewalks, cycle paths and parks and creating violence prevention policies (Malta et al., 2011). The participants acknowledged the availability of these outdoor facilities; however, they explained that avoidance of the sun and the risk of violence in the street were barriers to using them. The fear of urban violence as a barrier to exercise in Brazil has been reported previously (Morais et al., 2010). This suggests the need for a review of the violence prevention policies, provision of shade in outdoor facilities and increased awareness of indoor public leisure facilities.

Brazil has successfully decreased cigarette smoking among the population following increased advertisement bans, taxation, health warnings and treatment and support (Szklo et al., 2012). One participant suggested that this public health example should be followed to decrease junk food consumption, particularly prohibition of junk food advertisements in supermarkets and on the television. Dubois et al showed that banning the advertising of potato chips in the UK market can reduce the quantity sold by 10% (Dubois et al., 2018). The WHO recommends that an advertising ban should reduce exposure to the marketing of foods high in saturated fats, trans-fatty acids, salt or free sugars (World Health Organization, 2010).

An important finding was that weight loss treatment through the SUS is perceived to be less accessible due to lack of awareness of available treatments, longer referral periods, stringent criteria for surgery and a lack of staff such as nutritionists, who participants have found very useful in the weight loss process. These reasons led patients in the study to access weight loss treatment privately, available through employee benefits or out-of-pocket. Only one-quarter of the Brazilian population access private health insurance, meaning the majority of the population have reduced access to weight loss treatment (Santos et al., 2008).

The current study identified doubt in the success of treatment and fear of mental health problems and death following surgery as barriers to accessing treatment which has not been reported in previous Brazilian literature. This suggests the increased influence of inaccurate information and weight loss myths in Brazil on decision making of treatment and the need of accurate information dissemination. Participants identified the health system or health professionals as reliable sources of information in providing accurate information to achieve successful weight loss in comparison to the media. One participant suggested increasing the promotion of weight loss techniques through healthcare professionals in the community. This could be provided through community health workers promoting a healthy lifestyle during home visits (Macinko & Harris, 2015). However, previous literature have reported inaccurate information existing amongst healthcare professionals which indicates the need for adequate training and education to healthcare professionals (Stanford et al., 2018).

Unfortunately, potential harmful consequences of diets have been reported in the literature such as hunger, depressed mood, irritability, decreased sociability and binge eating which can eventually develop into bulimia nervosa (Yanovski et al., 2000). Additionally, anti-obesity medication can produce side-effects such as faecal incontinence, diarrhoea, flatulence and dyspepsia (MacDaniels & Schwartz, 2016). The most commonly used anti-obesity medications in Brazil are orlistat and sibutramine, with most participants mentioning the use of sibutramine, which is not available in the European and US markets due to its association with increased risk of cardiovascular events and strokes (De Carvalho et al., 2011; Kang & Park, 2012). Participants in this study reported some of the side effects described in the literature as a barrier. This highlights the importance of safety netting to ensure patients understand safe weight loss methods and side effects of treatment.

Mental health conditions are linked to obesity and can influence weight loss and weight gain (Janney et al., 2018; Scott et al., 2008). Additionally, mental health conditions such as depression are linked to reduced quality of sleep which some participants identified as a barrier to weight loss (Zhang et al., 2017). This is supported by further literature, reporting that sleep deprivation can increase food intake and appetite (St-Onge, 2017). These findings emphasize the importance of psychological treatment and change in mindset before commencing weight loss treatment and the crucial role of the psychologist in the multidisciplinary approach to weight loss. The participants explained that psychologists can increase patients’ self-esteem and will-power which are important facilitators. The literature have reported that the multidisciplinary approach, involving the psychologist and nutritionist and regular follow-up consultations are effective in facilitating weight loss, improving post-operative outcomes and maintaining motivation (Chaim et al., 2017). Therefore, healthcare professionals should encourage and remind patients to attend these crucial follow-up appointments and maintain a good relationship with patients by ensuring words of encouragement or guidance are not misinterpreted as criticism by patients.

### Facilitators

Eating habits such as consumption of healthy food, eating at regular intervals and chewing slowly are important for weight loss (Oliveira & Silva, 2014). Participants reported that this is facilitated by the gradual change in eating habits to prevent cravings and relapse to unhealthy eating habits. This supports previous findings that the inclusion of a small portion of a sweet snack can facilitate diet control (Metzgar et al., 2015). A change in eating habits can lead to social isolation due to avoidance of temptations of unhealthy food at social gatherings (Oliveira & Silva, 2014). Some participants identified the preparation of a packed meal at home to take to work or social gatherings as a facilitator to diet control and prevents social exclusion. Documenting eating habits is an accurate method to examine eating habits and participants identified it as a facilitator to weight loss, allowing for reflection and progression of lifestyle changes (Margetts & Nelson, 1997). Setting alarms as reminders to eat at regular intervals is another facilitator described by participants.

A cooperative food network consisting of local gardens to cultivate fruit and vegetables to be sold within the community at affordable prices was a suggestion made by one participant to facilitate a healthy lifestyle. These cooperative food systems aim to supply fresh and healthy food locally, keep money in the community and celebrate good food, culture and community. An example of a cooperative food network is the “Local Organic Food Co-ops Network” in Ontario, Canada which is a non-profit organization (Sumner et al., 2014). Following this example, strong leadership, education, training and information and cooperation among cooperatives, including financial support are essential factors for the success of a network and need to be considered if the initiative is done in Brazil.

To overcome the barriers, access to social capital is a crucial facilitator for weight loss motivation (S Palmeira et al., 2016). However, participants described opposing diets and goals within the household and social environment as a barrier to weight loss, increasing temptation and lack of commitment to a healthy lifestyle, supporting previous literature (Oliveira & Silva, 2014; S Palmeira et al., 2016). Venturini discussed that families should perceive themselves as an integral part to the weight loss process and eating habits should change for the whole family; thus, education to family members on their role in weight loss support is required (Venturini, 2000).

A novel finding of the study is the huge impact of expert patients in inspiring and motivating patients to lose weight. Expert patients allowed for visualization of the weight loss changes, either through pictures or in real life, and learning about the health, physical and social improvements experienced as well as the opportunity for questions to be answered. Shared social identity between obese patients is a facilitator to weight loss through sharing information, problems and celebrating patients’ successes (Tarrant et al., 2017). Social capital and community assets can facilitate lifestyle changes and be especially beneficial in communities with limited resources (Riley et al., 2015). The current study suggests that expert patients should be a component of social capital for health promotion and to be accessible through the media such as the internet and TV channels as well as through the health system and weight loss programs.

### Future implications

These findings may inform the development of further approaches to facilitate weight loss in individuals. For example, the mixed views on the cost of healthy food demonstrate the need for education on financially accessible healthy meals. The Brazilian government have published dietary guidelines for the population, including healthy meal options (Ministry of Health of Brazil, 2014). These should be made easily accessible, comprehensive and concise for the public. Prohibition of junk food advertisements in Brazil and improving affordability and safety of exercise facilities should be considered. Cooperative food networks using abandoned plots to cultivate fruit and vegetables which can be sold within the community should be considered. Increased education providing accurate information is required to avoid harmful and unsuccessful weight loss attempts. Information provided should include expert patients’ examples, the benefits of using a weight loss program with a multidisciplinary team, use of a food diary to control diets and time-management skills such as setting an alarm for mealtimes. The multidisciplinary approach to weight loss, especially the use of psychologists is essential, and a good relationship between healthcare professionals and patients is crucial to maintain trust and motivation for weight loss. In addition, local governments should continue to improve the environment and opportunities for physical activity, particularly violence prevention policies and provision of shade as identified in the study, to create healthy, active cities recommended by the WHO (Edwards & Tsouros, 2006; Malta et al., 2011). Future research should assess the effectiveness of education methods, particularly the use of expert patients and the quality of weight loss education and information in Brazil.

### Limitations

The study has some limitations. This was a cross-language qualitative study with the use of interpreters. Qualitative thematic analysis relies on deriving meaning from words and data, and some meanings may be lost in translation from Brazilian Portuguese to English leading to bias of misinterpretation (Braun & Clarke, 2006; Van Nes et al., 2010). Measures were taken to minimize this by a second interpreter reviewing the accuracy of translations and the use of redacted translation, meaning timings of the audio-recording being documented at regular intervals on the transcripts to allow for clarification if required. Due to lack of availability of one single interpreter, three interpreters were used during the interviews. To avoid potential inconsistencies in data collection, CM was present during all of the interviews and practiced and briefed the interpreters on asking questions. The use of a local interpreter provided benefits as they could explain cultural contexts to the researcher and provide insight into the meaning of responses. The BMI of CM and the interpreters were normal which may lead to social desirability bias (World Health Organisation, 1995). The normal BMI of the researcher may also affect the analysis and interpretation of the study; however, the primary researcher took a reflexive approach during the study and analysis triangulation was conducted to minimize this.

More women (Silva et al., 2011) participated in the study than men (World Health Organization, 2016) which may introduce under coverage bias and that the range of views may be limited and not generalizable to the whole population. However, the under representation of men in the study may be due to the higher proportion of women who are obese and the higher proportion of women seeking healthcare compared to men in Brazil (Pinheiro et al., 2002; World Health Organization, 2016). Participants covered all ranges of monthly household income except the highest range, indicating that the results can be generalized to the range of monthly household income covered in the study. However, participants were recruited from a private weight counselling clinic which may introduce sampling bias since only one quarter of Brazilians have access to private healthcare so the results may only be generalizable to the population that have access to private healthcare (Santos et al., 2008). Future studies should include more views from the highest and lowest household incomes and patients accessing the public healthcare system, as this study highlighted the importance of financial situation with weight loss and previous literature have reported that obesity is growing among the lowest socioeconomic group in Southeast Brazil (Sawaya et al., 1995).

### Conclusion

This study explored the barriers and facilitators to weight loss in adults who are overweight and obese in Southeast Brazil, contributing to the existing literature. Novel findings included the use of expert patients as health educators and the perceived lack of accessibility to weight loss treatment through the SUS as a barrier to weight loss. Barriers to a healthy lifestyle such as financial difficulties, time, junk food advertising and participants’ perceived lack of opportunity for safe outdoor exercise need to be addressed. Education was highlighted as an important facilitator and can be used to challenge these barriers and dispel myths on weight loss treatment, with expert patients being described as the most beneficial education method. At the individual level, successful weight loss requires a lifelong commitment, motivation and support from a multidisciplinary team, family and friends. At a societal level, removing the barriers and promoting the facilitators have the potential to achieve successful weight loss and a reduction in obesity rates in Brazil, improving health, social and economic consequences of the disease.

## Data Availability

The datasets used and analyzed during the current study are available from the corresponding author on reasonable request.

## Geolocation information

Key words: Barriers, Brazil, facilitators, obesity, qualitative, weight loss
